# Identification of a KPC Variant Conferring Resistance to Ceftazidime-Avibactam from ST11 Carbapenem-Resistant *Klebsiella pneumoniae* Strains

**DOI:** 10.1128/spectrum.02655-21

**Published:** 2022-04-13

**Authors:** Yuchen Wu, Xuemei Yang, Congcong Liu, Yanyan Zhang, Yan Chu Cheung, Edward Wai Chi Chan, Sheng Chen, Rong Zhang

**Affiliations:** a Department of Clinical Laboratory, Second Affiliated Hospital, Zhejiang University School of Medicine, Hangzhou, China; b Department of Infectious Diseases and Public Health, Jockey Club College of Veterinary Medicine and Life Sciences, City University of Hong Kong, Kowloon, Hong Kong; c State Key Lab of Chemical Biology and Drug Discovery, Department of Applied Biology and Chemical Technology, Hong Kong Polytechnic University, Hung Hom, Hong Kong; University of Pittsburgh School of Medicine

**Keywords:** KPC, ceftazidime-avibactam, *Klebsiella pneumoniae*

## Abstract

A novel Klebsiella pneumoniae carbapenemase (KPC) variant, KPC-93, was identified in two Klebsiella pneumoniae clinical isolates from a patient from China treated with ceftazidime-avibactam. KPC-93 possessed a five-amino-acids insertion (Pro-Asn-Asn-Arg-Ala) between Ambler positions 267 and 268 in KPC-2. Cloning and expression of the *bla*_KPC-93_ gene in Escherichia coli, followed by determination of minimum inhibitory concentration (MIC) values and kinetic parameters, showed that KPC-93 exhibited increased resistance to ceftazidime-avibactam, but a drastic decrease in carbapenemase activity. Our data highlight that a KPC variant conferring resistance to ceftazidime-avibactam could be easily induced by ceftazidime-avibactam treatment and that actions are required to control dissemination of these determinants.

**IMPORTANCE** Ceftazidime-avibactam (CZA) is a novel β-lactam/β-lactamase inhibitor combination with activity against serine β-lactamases, including the Ambler class A enzyme KPC. However, during recent years, there have been increasing reports of emergence of new KPC variants that could confer resistance to CZA. This has limited its clinical application. Here, we reported a new KPC variant, KPC-93, that could confer CZA resistance. KPC-93 possessed a five-amino-acids insertion (Pro-Asn-Asn-Arg-Ala) between Ambler positions 267 and 268 in KPC-2. Our findings have revealed the potential risk of *bla*_KPC_ gene mutations associated with CZA exposure over a short period of time.

## INTRODUCTION

Carbapenem-resistant *Enterobacteriaceae* (CRE), especially carbapenem-resistant Klebsiella pneumoniae (CRKP), have been highlighted as an urgent threat to global public health by virtue of the high fatality rates and financial burden of infections (1, 2). A retrospective cohort in Brazil showed that the overall 30-day mortality rate of hospitalized adult patients with bloodstream infections (BSI) caused by CRKP was up to 60% (3, 4). The dissemination and circulation of carbapenem resistance in K. pneumoniae strains further complicates clinical practice, leaving few treatment options, such as tigecycline and colistin, which are last-line defenses against CRE infections (5, 6). However, their efficacy has been significantly compromised by their toxicity and the rapid emergence and continuous transmission of tigecycline- and colistin-resistance determinants, such as the plasmid-borne resistance-nodulation-division (RND) efflux pump TmexCD1-ToprJ1, flavin-dependent monooxygenase Tet(X) variants, and movable colistin resistance (*mcr*) genes (6–9).

Ceftazidime-avibactam (CZA), a novel β-lactam antibiotic-β-lactamase inhibitor combination, has become a potential alternative to tigecycline and colistin against complicated intra-abdominal urinary tract infections and hospital-acquired pneumonia caused by CRKP strains, especially those producing Klebsiella pneumoniae carbapenemase (KPC), the most predominant class A serine β-lactamase conferring carbapenem resistance among K. pneumoniae strains globally (10, 11). Avibactam (AVI) is a potent diazabicyclooctenone (DBO) inhibitor of a wide range of β-lactamases, including many class A and C and some class D serine β-lactamases (SBLs) (12). When combined with ceftazidime, AVI protects ceftazidime from degradation by serine β-lactamases through covalent acylation of the SBL active sites (10). Whereas, since the approval of clinical use from FDA in 2015, CZA resistance has been increasingly reported, mainly caused by β-lactamase mutations (13–15). At the same time, according to the NCBI Reference Sequences (RefSeq) database (https://www.ncbi.nlm.nih.gov/pathogens/refgene/#KPC), the number of novel KPC variants has soared unprecedentedly, including many special variants that could confer resistance to enzyme inhibitors, such as KPC-8, -30, -31, -32, -40, -41, -50, -57, -58, -71, -74, and -78. Some KPC variants confer resistance to both inhibitors and extended-spectrum β-lactamases, such as KPC-14, -28, -33, -46, -51, -52, -53, -66, -72, -73, -79, and -82. Such a high number of variants may severely limit anti-infection therapeutic efficacy in clinical settings and pose significant threats to human health.

In this study, we identified a new KPC variant, KPC-93, from two clinical CZA-resistant K. pneumoniae strains isolated from a patient after intermittent administration of CZA for about 40 days. Its resistance profiles and mechanisms of resistance were investigated. Our findings have increased the diversity of KPC and shown the treatment dilemma for KPC-producing CRE strains.

## RESULTS

### Clinical strains and medical history.

**FIG 1:**
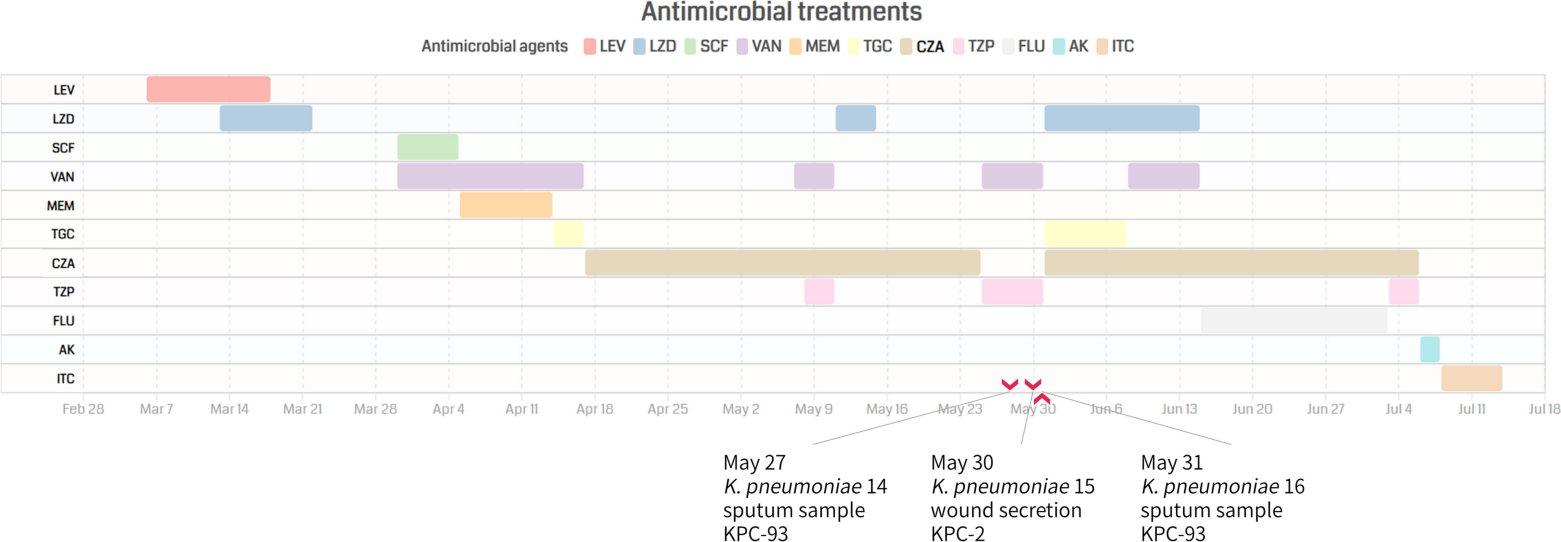
Antimicrobial treatments of the patient and isolation of K. pneumoniae strains.

In March 2021, a 61-year-old man was admitted to the Second Affiliated Hospital of Zhejiang University with a surgical site infection (SSI) after a lumbar discectomy. The patient experienced recurrent infection during treatment. After administration of CZA for about 25 days (2.5g q8h/d) (Fig. 1), three CRKP strains were isolated from sputum and secretion samples, including two CZA-resistant strains (K. pneumoniae 14 and 16) from sputum samples and one CZA-susceptible strain (K. pneumoniae 15) from a secretion sample. After 4 weeks of treatment, the patient improved, and all his symptoms resolved. None of the CZA-resistant strains were detected or isolated from his subsequent samples. Broth microdilution susceptibility testing showed that strains K. pneumoniae 14, 15, and 16 were resistant to aztreonam, amoxicillin, cefotaxime, ceftazidime, cefmetazole, cefepime, meropenem, ertapenem, piperacillin-tazobactam, cefoperazone/sulbactam, ciprofloxacin, and amikacin, but susceptible to polymyxin B and tigecycline and intermediate to imipenem (Table 1). Furthermore, strains K. pneumoniae 14 and 16 exhibited increased CZA resistance but decreased carbapenemem resistance compared to strain K. pneumoniae 15.

**TABLE 1:** MICs of antimicrobial agents for K. pneumoniae clinical isolates and E. coli TOP10 transformants

Antimicrobial agent^*a*^	MIC (μg/mL)							
	K. pneumoniae			E. coli				
	14 (KPC-93)	15 (KPC-2)	16 (KPC-93)	TOP10 (pTOPO-KPC-2)	TOP10 (pTOPO-KPC-41)	TOP10 (pTOPO-KPC-93)	TOP10 (pTOPO)	ATCC 25922
AMP	>128	>128	>128	>128	>128	>128	8	8
ATM	>128	>128	>128	256	4	4	<0.25	<0.25
AMX	>128	>128	>128	>128	>128	>128	16	8
AMC	>128/2	>128/2	>128/2	>128/2	128/2	>128/2	8/2	8/2
CTX	>128	>128	>128	16	16	8	<0.25	0.5
CAZ	>128	>128	>128	32	128	256	1	<0.25
CZA	64/4	1/4	32/4	0.5/4	16/4	64/4	0.5/4	<0.25/4
FEP	>64	>64	>64	ND^*b*^	ND	ND	ND	ND
CMZ	64	>128	64	ND	ND	ND	ND	ND
TZP	>256/4	>256/4	>256/4	ND	ND	ND	ND	ND
SCF	256/128	256/128	256/128	ND	ND	ND	ND	ND
IMP	2	16	2	4	1	0.5	<0.25	0.5
MEM	8	128	16	2	0.125	0.06	<0.25	<0.25
ETP	32	>128	32	2	0.25	0.25	<0.25	<0.25
CIP	>32	>32	>32	ND	ND	ND	ND	<0.5
AK	>128	>128	>128	ND	ND	ND	ND	2
PB	1	1	1	ND	ND	ND	ND	<0.5
TGC	1	1	1	ND	ND	ND	ND	<0.5

a AMP, ampicillin; ATM, aztreonam; AMX, amoxicillin; AMC, amoxicillin-clavulanate; CTX, cefotaxime; CAZ, ceftazidime; CZA, ceftazidime-avibactam; FEP, cefepime; CMZ, cefmetazole; TZP, piperacillin-tazobactam; SCF, cefoperazone/sulbactam; IMP, imipenem; MEM, meropenem; ETP, ertapenem; CIP, ciprofloxacin; AK, amikacin; PB, polymyxin B; TGC, tigecycline.

b ND, not detected.

### Genomic characterization.

To better understand the genetic environment of this difference, the complete genomes of strains K. pneumoniae 14, 15, and 16 were obtained by whole-genome sequencing (Table 2). The genome of strain K. pneumoniae 14 was composed of a 5,474,052-bp chromosome and four plasmids with sizes of 197,415, 133,129, 87,095, and 11,970 bp, respectively. The genome of strain K. pneumoniae 15 was composed of a 5,477,020-bp chromosome and four plasmids with sizes of 197,415, 133,114, 87,095, and 11,970 bp, respectively. The genome of strain K. pneumoniae 16 was composed of a 5,474,051-bp chromosome and four plasmids with sizes of 197,415, 133,129, 87,095, and 11,970 bp, respectively. Pairwise single-nucleotide polymorphism (SNP) analysis for these three strains showed that their core genomes differed only by a few SNPs (*n* ≤ 4). Furthermore, the plasmidomes of these three strains were almost the same, suggesting that these strains might originate from a single clone. These strains were all found to belong to sequence type (ST) 11 based on multi-locus sequence typing (MLST), and to the KL64 and O2v1 serotypes by Kaptive based on capsule and lipid synthesis loci. Analysis of acquired antimicrobial resistance identified 13 antimicrobial-resistance genes encoding resistance to β-lactams, aminoglycosides, fluoroquinolones, tetracyclines, chloramphenicol, sulfonamide, and trimethoprim (Table 2). In addition, *in silico* plasmid replicon typing indicated that these plasmids belonged to the FIBk/HI1B, FII/R, FII, and ColRNAI incompatibility (Inc) groups, respectively (Table 2).

**TABLE 2:** Genetic characterization of strains K. pneumoniae 14, 15, and 16

Strain	Plasmid or chromosome	Size (bp)	Resistance and virulence genes	Inc type^*a*^
K. pneumoniae 14	Chromosome	5,474,052	*bla*_SHV-182_, *aadA2*, *sul1*	
	pKP14_Vir	197,415	*iucABCDiutA*, *rmpA and rmpA2*, *iroN*, *peg344*	FIBk, HI1B
	pKP14_KPC	133,129	*bla*_CTX-M-65_, *bla*_TEM-1_, *bla*_KPC-93_, *bla*_SHV-12_	FII, R
	pKP14_res	87,095	*bla*_LAP-2_, *qnrS1, tet*(*A*), *catA2*, *dfrA14*, *sul2*	FII
	pKP14_col	11,970		ColRNAI
				
K. pneumoniae 15	Chromosome	5,477,020	*bla*_SHV-182_, *aadA2*, *sul1*	
	pKP15_Vir	197,415	*iucABCDiutA*, *rmpA and rmpA2*, *iroN*, *peg344*	FIBk, HI1B
	pKP15_KPC	133,114	*bla*_CTX-M-65_, *bla_TEM_*_-1_, *bla_KPC_*_-2_, *bla_SHV_*_-12_	FII, R
	pKP15_res	87,095	*bla_LAP_*_-2_, *qnrS1*, *tet*(*A*), *catA2*, *dfrA14*, *sul2*	FII
	pKP14_col	11,970		ColRNAI
				
K. pneumoniae 16	Chromosome	5,474,051	*bla_SHV_*_-182_, *aadA2*, *sul1*	
	pKP16_Vir	197,415	*iucABCDiutA*, *rmpA and rmpA2*, *iroN*, *peg344*	FIBk, HI1B
	pKP16_KPC	133,129	*bla_CTX_*_-M-65_, *bla_TEM_*_-1_, *bla_KPC_*_-93_, *bla_SHV_*_-12_	FII, R
	pKP16_res	87,095	*bla_LAP_*_-2_, *qnrS1*, *tet*(*A*), *catA2*, *dfrA14*, *sul2*	FII
	pKP14_col	11,970		ColRNAI

a Inc, incompatibility group.

**FIG 2:**
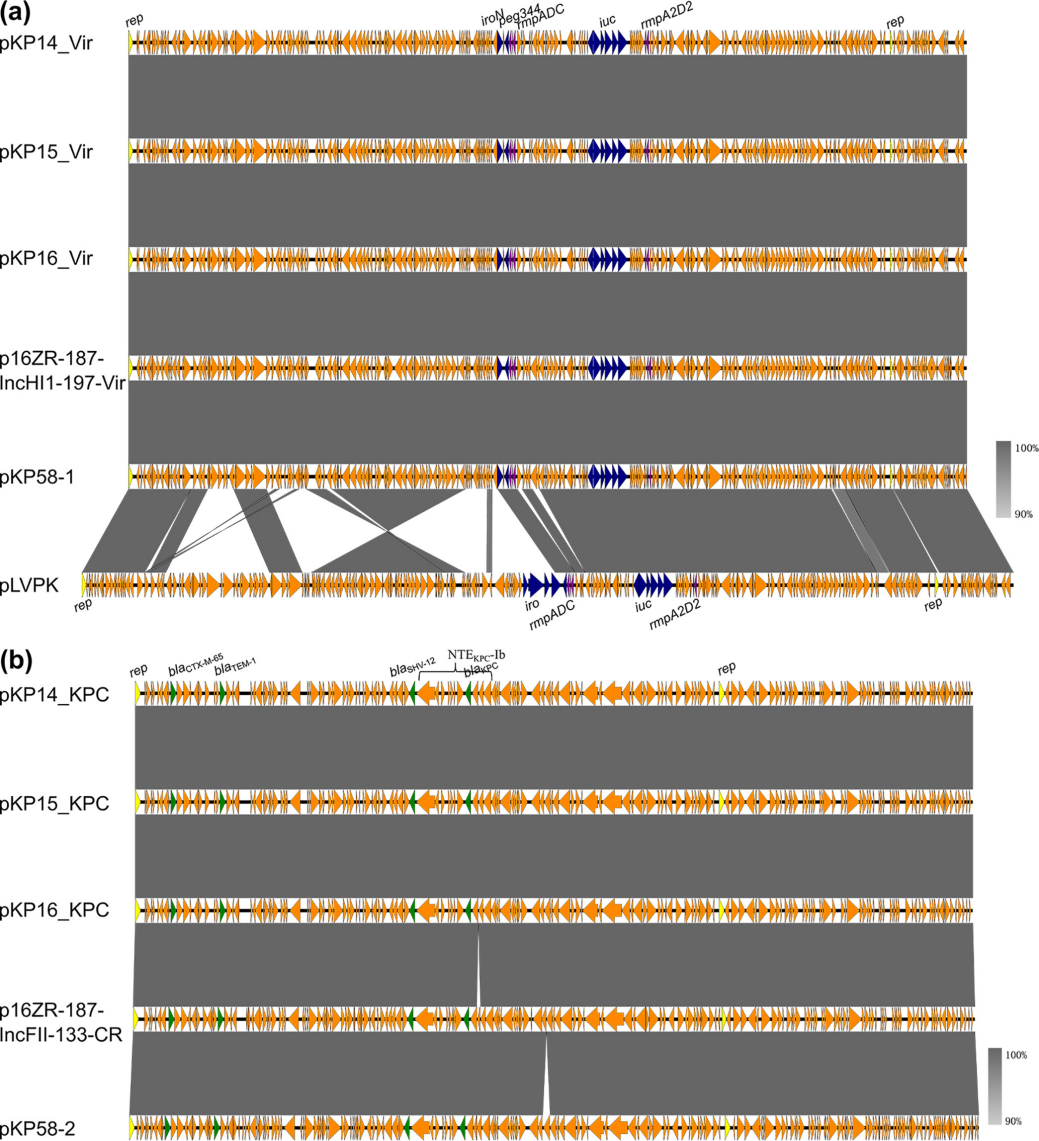
Alignment of virulence plasmids (a) and KPC plasmids (b) with similar structures in K. pneumoniae strains 14, 15, and 16 using Easyfig. Colored arrows indicate open reading frames and gray shading indicates regions of shared homology among different elements.

The 197,415-bp IncFIBk/HI1B, comprising 241 predicted protein-coding genes with a GC content of 50.05%, was identical among these three strains. Being highly similar (100% coverage and 99.99% identity) to plasmids pKP58_1 (GenBank accession no. CP041374.1) and p16ZR-187-IncHI1-197-Vir (GenBank accession no. MN182749.1), this plasmid harbored the virulence genes *iucABCDiutA*, *rmpA*, and *rmpA2*, and was designated pKP14_Vir (Table 2, Fig. 2a). Strain K. pneumoniae 15 was found to harbor the *bla*_KPC-2_ gene, which was located in a 133,114-bp IncFII/R plasmid with a GC content of 53.34% and comprised of 168 predicted protein-coding genes; this plasmid was designated pKP15_KPC (Table 2, Fig. 2b). The *bla*_KPC-2_ gene was carried on the NTE_KPC_-Ib transposon. Being highly similar (100% coverage and 99.99% identity) to plasmids pKP58_2 (GenBank accession no. CP041375.1) and p16ZR-187-IncFII-133-CR (GenBank accession no. MN182746.1), this plasmid also harbored the resistance genes *bla*_CTX-M-65_, *bla*_TEM-1_, and *bla*_SHV-12_ (Fig. 2b). Strains K. pneumoniae 14 and 16 also harbored this plasmid, but with a 15-bp insertion in *bla*_KPC-2_ gene, which might be the cause of CZA resistance in K. pneumoniae strains 14 and 16 (Fig. 2b).

### Identification of a novel KPC variant.

**FIG 3:**
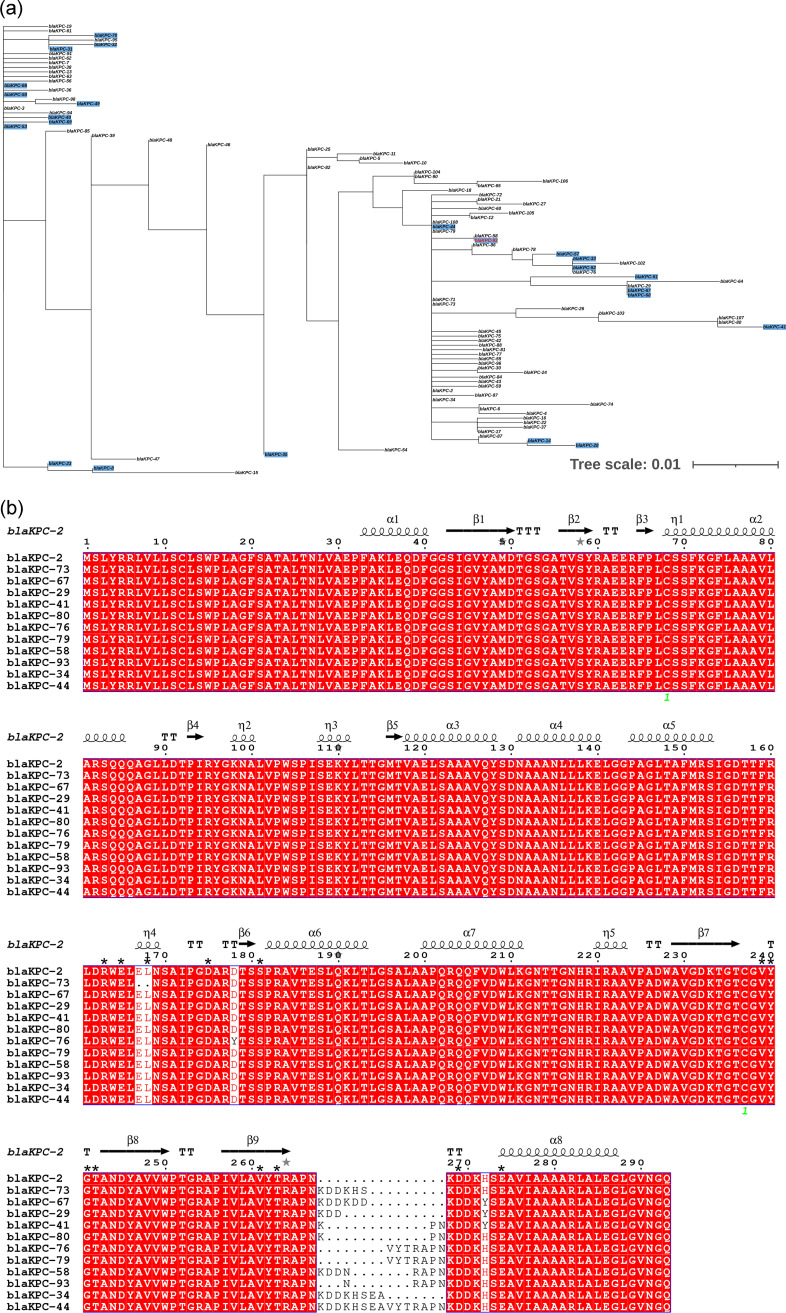
(a) Phylogenetic tree of KPC-93 and KPC variants. Blue background denotes CZA resistance. (b) Sequence alignment of KPC variants with insertion mutations at Ambler positions 267 and 268 in KPC-2. Key residues known to be implicated in CZA resistance are indicated by inverted black triangles.

Sequence analysis indicated that the novel allele possessed a five-amino-acids insertion (Pro-Asn-Asn-Arg-Ala) between Ambler positions 267 and 268 in KPC-2, and it was designated KPC-93 (Fig. 3c). The number of KPC carbapenemase variants has increased rapidly since 2020 (Fig. 3a) (16). So far, 84 KPC alleles have been deposited in the GenBank database. Among these, 22 have been demonstrated to confer CZA resistance, and the key residues known to be implicated in CZA resistance of KPC included positions R164, W165, L169, D179, V240, Y241, G242, T243, P266, D271, and E276 (Fig. 2b). Alignment of all KPC alleles indicated that KPC-29, -34, -41, -44, -58, -67, -73, -76, -79, and -80 showed insertion mutations in this site (Fig. 3c). Among these, KPC-41, -44, and -67 have been demonstrated to confer CZA resistance. (14, 17, 18).

### Characterization of KPC-93.

**FIG 4:**
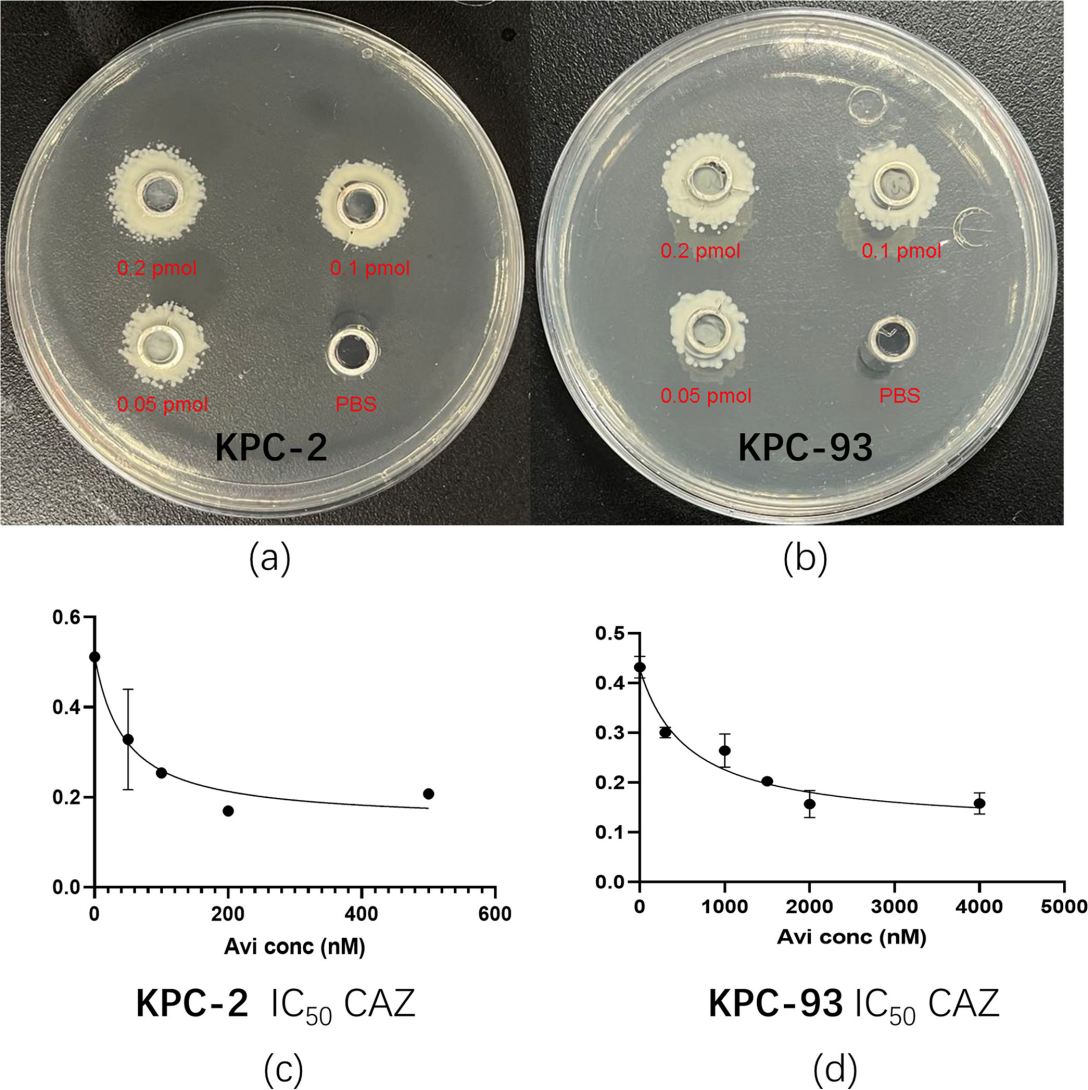
The hydrolysis and *K_i_* values of KPC-2 and KPC-93 against CAZ. (a) Hydrolysis of KPC-2 against CAZ. (b) Hydrolysis of KPC-93 against CAZ. (c) *K_i_* values of KPC-2. (d) *K_i_* values of KPC-93.

To confirm whether bla_KPC-93_ can mediate resistance to CZA, the DNA fragment encoding bla_KPC-93_ containing its natural promoter was amplified, ligated into vector pCR-BluntII-Topo, and transformed to E. coli strain TOP10. Constructs carrying *bla*_KPC-2_ and *bla*_KPC-41_ were also included for comparison. The MIC of CZA against E. coli strain TOP10 carrying *bla*_KPC-93_ was 64/4 μg/mL, a 128-fold increase compared that against *bla*_KPC-2_ and a 4-fold increase compared that against *bla*_KPC-41_. The MIC of imipenem against E. coli strain TOP10 carrying *bla*_KPC-93_ was shown to be decreased 8-fold compared that against *bla*_KPC-2_ and decreased 4-fold compared that against bla_KPC-41_ (Table 1). Furthermore, the kinetic constants of KPC-93 and KPC-2 for imipenem, meropenem, and ceftazidime could not be determined. The kinetic constants of KPC-2 and KPC-93 on nitrocefin were determined to be *K_m_* = 17.93 and 115.9 μM, respectively, and *k*_cat_ = 2.197 and 4.11 × 10^−4^ s^−1^, respectively (Table 3). The hydrolysis results indicated that both KPC-2 and KPC-93 can hydrolyze CAZ. The *K_i_* values of KPC-2 and KPC-93 for CAZ were determined. The IC50 of KPC2 was 45.1 nM and IC50 of KPC93 was 575 nM. (Fig. 4).

**TABLE 3:** Steady-state kinetic constants for hydrolysis of nitrocefin by purified KPC-2 and KPC-93

Enzyme	*K_m_* (μM)	*k*_cat_ (s^−1^)	*k*_cat_/*K_m_* (μM ^−1^ · s^−1^)
KPC-2	17.93	2.197	0.0123
KPC-93	115.9	4.11 × 10^−4^	3.55 × 10^−6^

**FIG 5:**
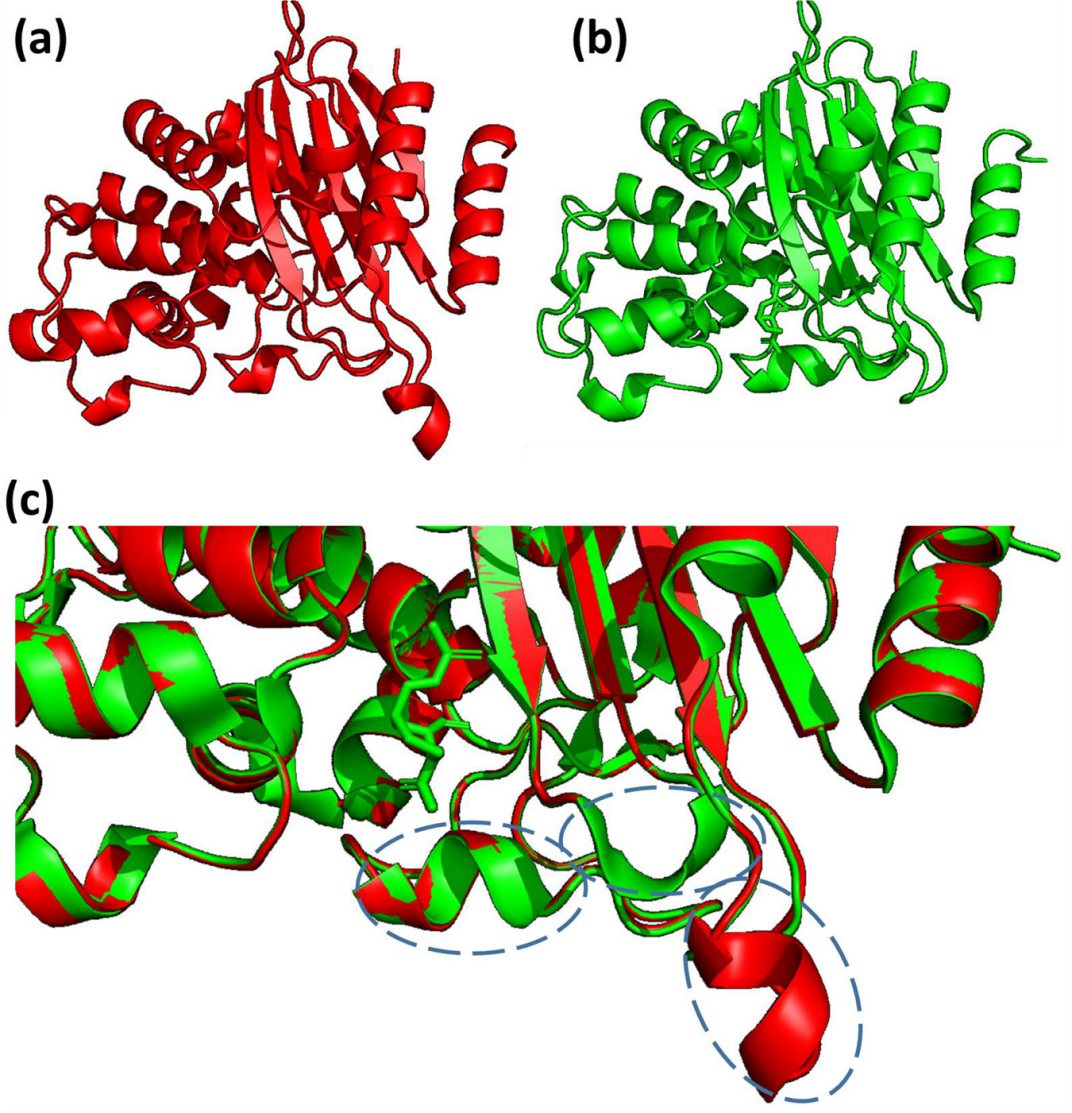
Structural model of KPC-93 using the crystal structure of KPC-2 complexed with avibactam (PDB ID: 4ZBE) as the template. (a) Modeled structure of KPC-93. (b) Structure of KPC-2. (c) Structure alignment of KPC-93 and KPC-2. Three major different regions were labeled.

Structure of KPC-93 was also modeled using structure of KPC-2 in complex with avibactam as the template (PDB ID: 4ZBE) and compared to that of KPC-2. We found three helical regions surrounding the active site of KPC-2 which exhibited confirmations different from those of KPC-93; this may cause conformational changes of KPC-93, leading to its resistance to inhibition by avibactam (Fig. 5).

## DISCUSSION

The global dissemination of KPC-producing K. pneumoniae (KPC-KP) has made ceftazidime-avibactam one of the few effective treatment alternatives against KPC-KP infections (11). However, sporadic reports of CZA resistance have risen since this novel β-lactam-β-lactamase inhibitor combination entered clinical routines in America in 2015 (15, 16, 19). China also approved its application in clinical routines on 21 May 2019 (13, 20). Here, we obtained two CZA-resistant K. pneumoniae strains producing KPC-93 carbapenemases with modest carbapenem resistance from sputum samples, and one carbapenem-resistant but CZA-susceptible K. pneumoniae strain carrying *bla*_KPC-2_ gene from an excretion sample, from the same patient in succession in China. Our data indicated that the novel variant of KPC-2, achieved through passage under the selection pressure of CZA, has contributed to augmented resistance to this antibiotic and reduced carbapenem resistance. This corroborated results from previous research which indicated that mutations in *bla*_KPC_ leading to CZA resistance have been linked to reversion to carbapenem susceptibility (15, 20).

There are three principal resistance mechanisms for KPC-KP strains to CZA, as reported, including co-production of metallo-β-lactamases, increased expression of KPC carbapenemases with porin mutations, and amino acid substitutions/deletions/insertions at pivotal loci of KPC carbapenemases, which was the leading cause of CZA resistance in KPC-KP strains worldwide (13, 16, 21). The Ω loop, with amino acid positions from 164_Arg_ to 179_Asp_, embraces the core of the active site of KPC β-lactamase and is vulnerable to mutations relevant to CZA resistance, especially 169_Leu_ and 179_Asp_ (15, 20, 22). The number of novel isoforms of KPC carbapenemases has increased rapidly since 2020 (16). So far, 98 mutant alleles of *bla*_KPC_ have emerged and 31 of them were classified as “inhibitor-resistant” according to the NCBI reference gene catalog. Most CZA-resistant KPC variant enzymes contained mutations at either of the loci mentioned above; most frequently at D179Y, followed by L169P, D179N, D179A, and L169Q (16, 20, 22–24).

Currently, no concentrated and epidemic CZA resistance mechanisms have been discovered in China in addition to the co-production of metallo-β-lactamases in KPC-KP strains (13). Shen et al. (25) found that increased copy number and gene expression of the wild-type *bla*_KPC-2_ combined with OmpK35 deficiency mediated CZA resistance in KPC-KP isolates in Shanghai. Xu et al. (26) reported that a novel *bla*_CMY-172_ AmpC β-lactamase was tightly associated with reduced CZA susceptibility in Zhejiang province. Additionally, CZA-resistant KPC-KP strains with KPC-2 variants KPC-33, KPC-51, and KPC-52, have been detected in Shanghai, Beijing, and Henan provinces (16, 20, 27).

In this study, we report the first case of KPC-93 carbapenemase-producing, ST11-type K. pneumoniae strains conferring resistance to CZA after exposure to CZA for 39 days and verified the one-way conversion from KPC-2 to KPC-93. It is worth noting that KPC-93 can reverse carbapenem resistance, like other variants. The low MIC values of carbapenems against K. pneumoniae harboring KPC-2/KPC-3 variants made them readily neglected, and the undetectable properties of some variants by NG-Test CARBA5 has further limited their early detection. Even more concerning is that the number of KPC variants is growing rapidly. The sum of newly identified KPC variants within the last 2 years has exceeded that of the past 2 decades. Various variants may create a more complex situation for drug applications, leading to higher risks to human health in the future.

In conclusion, because KPC-2-producing K. pneumoniae strains are highly prevalent in China and in other countries, it is vital for clinicians to monitor the susceptibility of these strains to CZA and adjust the dosage of CZA and therapeutic regimens to prevent therapy failure and the wide spread of CZA-resistant strains.

## MATERIALS AND METHODS

### Bacterial isolation and phenotypic characterization.

K. pneumoniae strains were isolated from clinical samples in a hospital located in Zhejiang, China in 2021. Strains K. pneumoniae 14 and 16 were isolated from sputum samples and strain K. pneumoniae 15 was isolated from a wound secretion from the same patient. Antimicrobial susceptibility was determined by the broth microdilution method using custom plates (DL Biotech, China) following Clinical and Laboratory Standards Institute (CLSI) guidelines (28) with 18 antimicrobial agents, including ampicillin, aztreonam, amoxicillin, amoxicillin-clavulanate, cefotaxime, ceftazidime, ceftazidime-avibactam, cefmetazole, cefepime, imipenem, meropenem, ertapenem, piperacillin-tazobactam, cefoperazone/sulbactam, ciprofloxacin, amikacin, polymyxin B, and tigecycline. All tests were performed in duplicate and each test included three biological replicates. The breakpoint of tigecycline was interpreted according to Food and Drug Administration (FDA) guidelines (susceptible MIC, ≤2 mg/L; intermediate MIC, 4 mg/L; resistant MIC, ≥8 mg/L). The breakpoint of cefoperazone/sulbactam was interpreted according to the CLSI breakpoint of cefoperazone for Enterobacteriaceae.

### Whole-genome sequencing and bioinformatic analysis.

Genomic DNA was extracted using the PureLink Genomic DNA Minikit (Invitrogen, USA) according to the manufacturer’s instructions and sent to Novogene (China) for whole-genome sequencing using the Illumina NovaSeq 6000 platform. Genomic DNA was also subjected to the long-read Nanopore MinION platform after being treated with a supplementary sequencing kit (Oxford Nanopore Technologies, Oxford, United Kingdom). MinION libraries were prepared using the SQK-RBK004 nanopore sequencing kit according to the manufacturer’s instructions. The library was then added to a MinION flow cell (R9.4.1) and sequenced. Both short and long reads were *de novo* hybrid assembled using Unicycler v0.4.8 (29). Assembled genome sequences were annotated with RAST v2.0 (30). MLST was determined by using Kleborate software based on the types of genetic variations in the seven housekeeping genes (31). Capsular typing on the assembled sequences was performed using Kaptive (32). Virulence genes were identified by searching against the BIGSdb Klebsiella genome database (https://bigsdb.pasteur.fr/klebsiella/). The BLASTn command lines, with an 80% coverage and identity cutoff, were used to map genome sequences against antibiotic resistance genes, insertion sequences, and plasmid replicons. The resistance genes and plasmid replicon databases were obtained from the Center for Genomic Epidemiology (http://www.genomicepidemiology.org/). Alignment of plasmids with similar structures was performed using Easyfig_win_2.1 (33).

### KPC variant identification and phylogenetic analysis.

The protein sequences of 98 total KPC variants (KPC-2 to KPC-108) deposited in the NCBI GenBank database were downloaded. The genome sequences of strains K. pneumoniae 14, 15, and 16 were subjected to BLAST against all KPC variants, using blastx to identify KPC alleles. The KPC-2 allele was identified in strain K. pneumoniae 15, while a novel KPC-2 variant, KPC-93, was identified in strains K. pneumoniae 14 and 16. The protein sequences of KPC-93 and other KPC variants were aligned using the program Clustal Omega (34) and the final output was submitted to RAxML for phylogenetic analysis using a maximum-likelihood method (35). The resulted phylogenetic tree was further modified by iTOL (36). The aligned sequences were also subject to analysis by ESPript 3.0.58 (37).

### Cloning, expression, and purification of carbapenemases KPC-2, KPC41, and KPC-93.

The *bla*_KPC-2_ gene was amplified using the primers KPC2-all-fw and KPC2-all-rev. The PCR product was then ligated into vector pCR-BluntII-Topo (Invitrogen, USA) and the resulting plasmid, pTOPO-KPC-2, was further transformed into Escherichia coli strain TOP10. The *bla*_KPC-41_ and *bla*_KPC-93_ genes were obtained by site-directed mutagenesis, using the *bla*_KPC-2_ gene as the template, by the GeneArt Site-Directed Mutagenesis System (Invitrogen, USA) according to the manufacturer’s instructions.

Carbapenemases KPC-2, KPC41, and KPC-93 were purified from E. coli TOP10 strains containing plasmids pTOPO-KPC-2, pTOPO-KPC-41, and pTOPO-KPC-93, respectively. Briefly, bacteria were cultured overnight at 37°C in Luria broth with kanamycin (50 μg/mL) and pelleted by centrifuge at 4,500 × *g* for 30 min. Bacterial cells were then resuspended in 50 mM morpholine ethanesulfonic acid (MES) buffer (pH 5.5) and disrupted by sonication. After centrifuging at 30,000 × *g* for 1 h, the supernatant containing the enzymes was collected. Purification was performed in two steps. First, the supernatant was loaded onto a HiTrap Q HP column (GE Healthcare, Germany) which was preequilibrated with MES buffer and eluted by continuously increasing salt concentration (NaCl) from 0 to 1 M. Next, the collected fractions containing target proteins were concentrated using Amicon Ultra-15 Centrifugal Filter Units (Millipore, USA) and loaded onto a HiLoad 16/600 Superdex 75-pg size exclusion column (GE Healthcare, Germany) which was preequilibrated with 100 mM sodium phosphate (pH 7.0). Eluted proteins were concentrated, flash-frozen, and stored at −80°C. Purification was detected by SDS-PAGE.

### Enzymatic activity and kinetics characterization of carbapenemase KPC-93.

The antimicrobial susceptibility of E. coli TOP10 strains containing plasmids pTOPO-KPC-2, pTOPO-KPC-41, and pTOPO-KPC-93, respectively, was determined by the broth microdilution method. Steady-state kinetic parameters of carbapenemase KPC-93 were measured in 100 mM sodium phosphate (pH 7.0). The initial velocity of hydrolysis was determined using a UV-1900 UV-Vis spectrophotometer (Shimadzu, Japan). The hydrolysis rate of nitrocefin was determined using a wavelength of 482 nm and an absorption coefficient (Δε) of 15,000 (M^−1^ · cm^−1^).

### The hydrolysis of KPC-2 and KPC-93 against CAZ.

The hydrolysis of KPC-2 and KPC-93 against CAZ was determined. Briefly, a bacterial culture of a CAZ-susceptible K. pneumonia strain was suspended in saline and inoculated on agar plates containing 1 μg/mL CAZ. Different amounts of purified KPC-2 and KPC-93 proteins were added into the Oxford Cup. Phosphate-buffered saline was used as a negative control. The plates were cultured at 37°C for 16 h and bacterial growth was observed.

### Structure modeling of carbapenemase KPC-93.

Homology modeling of carbapenemase KPC-93 was performed using a crystal structure of KPC-2 in complex with avibactam as the template (PDB ID: 4ZBE) (38) on the Swiss-Model workspace (39). The models were visualized by PyMol under an academic/nonprofit license.

### Data availability.

Complete sequences of the chromosomes and plasmids of strains K. pneumoniae 14, 15, and 16 have been deposited in the GenBank database under BioProject number PRJNA779111. The novel KPC variant KPC-93 sequence has been deposited in the GenBank database under accession number MZ569034.1.
